# Large-scale retrospective analysis of methodological factors affecting pregnancy rates after embryo transfer for in vitro fertilization

**DOI:** 10.1097/MD.0000000000035146

**Published:** 2023-09-08

**Authors:** Mustecep Kavrut, Fulya Gokdagli Sagir, Zafer Atayurt

**Affiliations:** a Private Center, Fulya Academy, Istanbul Turkey; b Kolan International Hospital Gynecology, Obstetrics and IVF Center, Istanbul, Turkey; c Sisli Kolan International Hospital, IVF Center, Istanbul, Turkey.

**Keywords:** air bubble position, assisted reproductive technology, embryo transfer, pregnancy rate

## Abstract

This study aimed to investigate the impact of methodological factors on pregnancy rates after embryo transfer (ET) for in vitro fertilization. This retrospective cross-sectional study was conducted between September 2020 and April 2022. A total of 2048 patients who underwent ultrasonography-guided first frozen embryo transfer (FET) or a fresh ET cycle due to infertility were included in the study. The effects of age, ET protocol (frozen or fresh), preimplantation genetic testing, number of embryos transferred (NET), and embryo fundus distance on pregnancy rate were investigated. The mean age of pregnant patients (31.51 ± 5.28) was significantly lower than that of non-pregnant patients (35.34 ± 6.39) (*P* < .001). Multiple regression analysis showed that women with lower age (*P* < .001), higher NET (*P* < .001), higher embryo fundus distance (*P* < .001), FET (*P* < .001), and preimplantation genetic testing (*P* = .012) had a significantly higher likelihood of pregnancy. Appropriate transfer depth, younger age, euploid embryo transfer, FET, and a higher NET can increase the likelihood of pregnancy. However, multiple factors must be considered when deciding the best protocol for a particular patient, including patient preference, costs and timing.

## 1. Introduction

Infertility or impaired fertility is observed in at least 1 of every ten couples.^[1]^ Assisted reproductive technology (ART) is used worldwide as an inevitable method of infertility treatment.^[2]^ Many features of ART have advanced notably over the past 30 years. However, the embryo transfer (ET) procedure, a simple but crucial part of ART, remains relatively unchanged and a deciding factor for success.^[1]^

The aim of ET is to deliver optimal quality embryos to an appropriate position in the uterus to maximize the probability of implantation.^[3]^ However, several proven or possible clinical and embryological factors can affect ET success rates, including female age, body mass index, duration of infertility, infertility etiology, selected protocol, use of assistive devices such as ultrasonography (USG), preimplantation genetic testing (PGT), endometrial thickness, timing of ET, number of embryos transferred (NET), transfer catheter (TC) type, TC loading technique, and blood, mucus, or bacterial contamination.^[4–9]^ Moreover, other factors affect ET outcomes are not known clearly, such as air bubble position (ABP), embryo fundus distance (EFD), use of frozen/thawed frozen embryo transfer (FET) or fresh ET, and bed rest after ET.^[8,10–12]^ In a recent study, age was shown to be the most important pre-cycle variable, and the number of cryopreserved embryos and NET were shown to be the most important in-cycle variable for pregnancy outcomes.^[13]^ Another study showed that protocol type, gonadotrophin preparations, previous fresh cycle outcomes, endometrial thickness, and the number of obtained oocytes or embryos are factors that affect pregnancy outcomes.^[14]^ Determining the factors affecting ET outcomes will increase positive pregnancy rates (PPR) and live birth rates.^[4]^ Although there are many studies on this subject, most have various limitations, including low patient count,^[14,15]^ limited age range,^[16]^ investigation of only a few factors (loading technique,^[9]^ endometrial thickness,^[17]^ ABP or EFD^[15,18]^), and inclusion of only particular patient groups.^[6,19,20]^

This study aimed to investigate the effect of age and various methodological factors on pregnancy rates after ET.

## 2. Material and methods

### 2.1. Study design

This retrospective cross-sectional study was conducted between September 2020 and April 2022 in the Department of Reproductive Endocrinology, Infertility, and Assisted Reproductive Technologies Center, Sisli Kolan International Hospital, Istanbul, Turkey.

### 2.2. Ethical approval

This study was approved by the Ethics Committee of the ISTUN University Faculty of Medicine (date: August 16, 2022, no: 2022-01). This study was conducted in accordance with the ethical standards of the Declaration of Helsinki and its amendments.

### 2.3. Study population

A total of 2048 patients who underwent USG-guided first FET or fresh ET cycle (in vitro fertilization [IVF] or intracytoplasmic sperm injection) due to female infertility were included in the study. The exclusion criteria were as follows: body mass index ≥25 kg/m^2^, male infertility, endometrial thickness <7 mm during ET, history of recurrent pregnancy loss, history of recurrent ET failure, endometriosis or adenomyosis, presence of congenital or acquired uterine anomalies, endocrine disease, suspected ovarian cancer, use of oral contraceptives or intrauterine device in the 6 months prior to ET, difficulty in TC or repeated transfer attempts, and presence of systemic disease that may affect pregnancy likelihood (diabetes mellitus, autoimmune disease, etc).

Difficult ET was defined as the presence of any of the following: countering greater resistance, time-consuming procedure, discomfort to the patient, need to change to a stiff catheter, additional instrumentation requirements, such as cervical dilatation, and detection of blood in the catheter.^[21]^

### 2.4. Laboratory and genetic analysis

Blood samples were collected from the antecubital vein for hormone level analyses. All measurements were performed in the Clinical Chemistry Department of Sisli Kolan International Hospital using routine devices (Abbott Architect i2000; Abbott Diagnostics, Illinois) according to the manufacturer’s recommendations.

Genetic analyses were performed at the genetic laboratory of our hospital. After blastocyst-stage frozen embryos were thawed, 5 to 10 cells were removed from the trophectoderm, whole chromosomes were analyzed using comprehensive chromosome screening technology, and only euploid embryos were used for ET.

### 2.5. Assisted reproductive technology procedures

#### 2.5.1. Ovarian stimulation and fertilization.

Controlled ovarian stimulation was started on days 2 or 3 of menstruation. Recombinant follicle-stimulating hormone (Gonal-F; Merck Serono, Germany) was administered daily at a dosage based on age, antral follicle count, serum anti-Müllerian hormone, follicle stimulating hormone (FSH), and luteinizing hormone levels. Follicular growth was monitored using serial transvaginal USG and serum FSH, luteinizing hormone, and estradiol levels. A gonadotropin-releasing hormone antagonist (Cetrorelix; Cetrotide; Merck Serono, Germany) was used for pituitary suppression when a prominent follicle reached 14 mm. Recombinant human chorionic gonadotropin (hCG) (Ovitrelle; Merck Serono Pharmaceuticals, Geneva, Switzerland) was used as a trigger for both normal and poor responders. A gonadotropin-releasing hormone analog was used as a trigger for hyperresponders to reduce the risk of ovarian hyperstimulation syndrome. Responder classification was based on age, baseline anti-Müllerian hormone or FSH levels, antral follicle count, and ART trial count.^[10,22]^ Oocytes were retrieved transvaginally 36 hours after the triggering. Standard intracytoplasmic sperm injection or IVF techniques were applied to fertilize oocytes. Finally, fertilized oocytes were stored in a culture medium with appropriate CO_2_, humidity, and temperature. Fresh ET was performed on day 5 of embryo development. The blastocysts for fresh ET were selected according to the Gardner classification.^[23]^ All freezing procedures were performed on women who subsequently underwent FET.

#### 2.5.2. Embryo scoring, vitrification, and thawing.

Blastocysts were scored before vitrification according to Gardner classification. Good- and top-quality blastocysts were vitrified on the morning of day 5 or day 6 with Kitazato vitrification media (Kitazato BioPharma Co. Ltd, Japan) using Cryotops (Kitazato BioPharma Co. Ltd) as carriers. Blastocysts were thawed using Kitazato warming media (Kitazato BioPharma Co. Ltd.) according to the manufacturer instructions. The embryos were first checked for survival 30 minutes after thawing for re-expansion, hatching, extensive cytoplasmic granulation, and presence of necrotic areas. Blastocysts with at least 80% re-expansion and vitality were transferred onto the same day.

#### 2.5.3. Endometrial preparation.

USG evaluation was performed on the second day of menstruation to assess the presence of ovarian cysts or other pelvic pathologies. An artificial cycles-FET hormonal replacement protocol with estradiol (Estrofem 2 mg; Novo Nordisk, Bagsvaerd, Denmark) from days 2 or 3 of menstruation was performed for endometrial preparation of all FETs.^[24]^ The USG was repeated on days 10 or 11, depending on the patient menstrual cycle length. We evaluated endometrial thickness, and endometrial pattern, and the presence of any spontaneously growing dominant follicles. When the endometrial thickness was ≥8 mm, Vaginal Natural Micronized Progesterone (Progestan 200 mg; Koçak, Turkey) was started (3 times per day). ET was performed after administering vaginal progesterone for a total of 5 days.

#### 2.5.4. Embryo transfer.

The NET was determined according to maternal age, ART indication, and the number and quality of embryos. All ETs were performed by an expert infertility physician guided by transabdominal USG. The ET procedure was performed as follows. The women were placed in the lithotomy position with a moderately full bladder. The cervix was visualized using a vaginal speculum. Mucus in the cervical canal was cleared with a sterile cotton swab dipped in the culture medium. Care was taken to maintain proper CO_2_, humidity, and temperature until the embryo was transferred to the TC. Embryos were loaded into a soft TC (Wallace Sure-Pro Ultra; CooperSurgical; Knardrupvej, Denmark) using the “three-drop technique” in the following manner: 25 μL culture media, 20 μL of air, 25 μL of culture media containing the embryo, 20 μL of air, and a small amount of additional culture media.^[25]^ First, an external catheter is placed in the cervical canal. Using a guide, the internal catheter was advanced through an external catheter into the cavity. Under transabdominal USG, the TC contends were emptied into the most suitable position in the uterus, and the distance of this localization to the fundus was measured and recorded. The time between loading and transfer was no more than a few seconds for any patient. To prevent uncontrolled uterine contractions, the tip of the TC was carefully controlled to prevent contact with the uterine fundus. Finally, TC was slowly withdrawn. To check for retained embryos and document the absence of any blood, mucus, or endometrial tissue, the catheter was inspected by an embryologist using a stereomicroscope. After the procedure, the patient was allowed to stand for 60 minutes.

### 2.6. Positive pregnancy definition

A positive pregnancy (PP) was defined as a blood β-hCG level of more than 20 mIU/mL on the 12th day after ET.^[26]^

### 2.7. Grouping of the cases

The patients were grouped according to pregnancy outcomes, ET protocol (fresh ET or FET), NET, and ABP. The women were divided into 6 groups according to ABP as follows: group 1 (location in the fundus), group 2 (between the fundus and mid-cavity), group 3 (mid-cavity), group 4 (between the mid-cavity and the cervix), group 5 (location around the cervix-internal os), and group 6 (transfers relocating towards the cervix after placement).

### 2.8. Statistical analysis

All analyses were performed using IBM SPSS Statistics for Windows (version 25.0 (IBM Corp., Armonk, NY), with a significance threshold of < .05 (*P* value). Histograms and Q-Q plots were used to determine whether continuous variables were normally distributed. Data were summarized as mean ± standard deviation for continuous variables and as frequency (percentage) for categorical variables. Continuous variables were analyzed using the independent sample *t* test. Categorical variables were analyzed using the chi-square test or Fisher-Freeman-Halton exact test. Multiple logistic regression analysis was performed to determine the significant factors independently associated with the PPR.

## 3. Results

The mean age of all women participating in the study was 32.58 ± 5.87 (range 17–53) years. Age in the group with positive pregnancy (PPG) was 31.51 ± 5.28 years, while the non-pregnant group (NPG) had a mean age of 35.34 ± 6.39 years (*P* < .001).

Overall PPR was 71.9%. The percentage of patients who underwent FET was significantly higher in the PPG group than in the NPG group (*P* < .001). In the NPG, the percentage of women in Groups 5 and 6 was significantly higher than that in the PPG group (*P* < .001). The percentage of women who underwent 1 ET was significantly higher in the NPG, whereas the percentage of women who underwent 2 ETs was higher in the PPG group (*P* < .001) (Table 1).

**Table 1:** Summary of variables with regard to pregnancy test result.

	Total (n = 2048)	Pregnancy test result		*P*
		Negative (n = 576)	Positive (n = 1472)	
Age	32.58 ± 5.87	35.34 ± 6.39	31.51 ± 5.28	<.001
Embryo transfer				
Fresh	167 (8.2%)	71 (12.3%)	96 (6.5%)	<.001
Frozen	1881 (91.8%)	505 (87.7%)	1376 (93.5%)	
PGT	236 (11.5%)	55 (9.5%)	181 (12.3%)	.080
Position				
1	603 (29.4%)	153 (26.6%)	450 (30.6%)	<.001
2	982 (47.9%)	273 (47.4%)	709 (48.2%)	
3	284 (13.9%)	72 (12.5%)	212 (14.4%)	
4	14 (0.7%)	7 (1.2%)	7 (0.5%)	
5	12 (0.6%)	8 (1.4%)	4 (0.3%)	
6	153 (7.5%)	63 (10.9%)	90 (6.1%)	
NET				
1	669 (32.7%)	226 (39.2%)	443 (30.1%)	<.001
2	1335 (65.2%)	335 (58.2%)	1000 (67.9%)	
3	43 (2.1%)	15 (2.6%)	28 (1.9%)	

Data are summarized as mean ± standard deviation for continuous variables and as frequency (percentage) for categorical variables.

NET = number of embryo transferred, PGT = pre-implantation genetic testing.

Multiple logistic regression analysis revealed that women with lower age (*P* < .001) and higher NET (*P* < .001) had a greater likelihood of PP, while women with further bubble position (higher EFD) (*P* < .001) had a lower likelihood of PP. Women with FET had a 1.938-fold higher likelihood of PP than those who underwent fresh ET (OR, 1.938; 95% CI, 1.365 2.751; *P* < .001). In addition, women with PGT had a 1.551-fold greater likelihood of PP than those who had not undergone PGT (OR, 1.551; 95% CI, 1.101–2.186; *P* = .012) (Table 2).

**Table 2:** Significant factors of the positive pregnancy test result, multiple logistic regression analysis.

	β coefficient	Standard error	*P*	Exp (β)	95.0% CI for Exp(β)	
Age	−0.121	0.009	<.001	0.886	0.870	0.903
Frozen embryo transfer	0.661	0.179	<.001	1.938	1.365	2.751
PGT	0.439	0.175	.012	1.551	1.101	2.186
Position (EFD)	−0.157	0.038	<.001	0.855	0.793	0.921
NET	0.449	0.105	<.001	1.567	1.277	1.924
Constant	3.910	0.401	<.001	49.882		

Nagelkerke *R*^2^ = 0.155.

CI = confidence interval, EFD = embryo-fundus distance, NET = number of embryos transferred, PGT = pre-implantation genetic testing.

## 4. Discussion

IVF is being increasingly used as a treatment option for various types of infertility and yields high success rates. However, the chances of successful pregnancy using this technique can be affected by many factors.^[4,14]^ In the present study, older age, higher EFD, and air bubble relocation towards the cervix had negative effects, whereas the use of FET, PGT, and higher NET had positive effects on PPR in women who underwent ET.

Through technological developments, we are now able to view the exact position of the TC tip and place the embryo in an ideal position using USG guidance.^[10,27]^ However, the effects of uterine ABP on pregnancy outcomes remain controversial.^[3,7]^ In this study, the PPR was 71.9%. This rate was higher than that reported in previous studies assessing β-hCG levels.^[28,29]^ Our study also showed that PPR was reduced when the location of ABP was far from the fundus, and a systematic review noted that embryo location influenced PPR, and that the highest rates of pregnancy were achieved when the embryo was placed in the upper or middle region of the uterine cavity, at least 1 cm from the fundus.^[7]^ Wang et al reported that both PPR and live birth rates were higher when the EFD was ≥10 mm and ≤20 mm.^[11]^ Yang et al showed that when the EFD is >19 mm, the clinical pregnancy likelihood decreases by 16% for each 1 mm increase.^[18]^ A prospective analysis of on 1184 IVF cycles showed that an EFD between 5 and 15 mm resulted in higher pregnancy and implantation rates than an EFD > 15 mm.^[30]^ However, it is critical to note that the site of the air bubble does not conclusively identify the site of embryo implantation.^[3]^ Many factors can affect this situation, such as uterine contractility, uterin position (retroverted, anteverted, horizontal), injection speed, density difference between the embryo and air bubble, change in the surface tension of the air bubble, and cavity pressure.^[3,31]^ As observed with USG, ≥90% of the transferred air bubbles did not move into the uterus after ET. In addition, air bubbles appeared immediately after the transfer in approximately 80% of the gestational sac.^[10]^ As noted in a prospective study, ABP within the first 60 minutes of ET appeared to predict the implantation site in approximately half of the cases.^[31]^ Our study showed that relocation of the ABP to the cervix is associated with reduced PPR. Similarly, Ozcan et al stated that the displacement of air bubbles after ET towards the cervical canal could be a poor prognostic factor for PPR. The results of our study support the argument that ABP should be close to the fundus and away from the cervix. We also suggest that optimizing EFD and taking measures to prevent ABP displacement may increase the likelihood of successful implantation and by extension, PPR.

The pregnancy success rate of FET compared to that of fresh ET remains controversial.^[1]^ While there are studies that argue that the ultimate success of FET is not different from that of fresh ET,^[11,32]^ there are also studies that have reached opposite conclusions.^[12,33]^ In our study, FET was found to be factor that positively affect the PPR. In a multicenter retrospective study of 2910 women who underwent IVF-ET, the overall implantation and ongoing pregnancy rates were significantly higher in the freeze-only ET group than in the fresh ET group.^[34]^ Another retrospective study demonstrated that biochemical pregnancy, clinical pregnancy and live birth rates were significantly higher in the FET group than in the fresh ET group among women aged ≥35 years.^[35]^ Conversely, in a retrospective study, a significant increase was found in the implantation rate with fresh cycles outperforming all frozen cycles.^[19]^ In addition to the many advantages of FET,^[4,34]^ its positive effect on PPR, detected in most studies and our study, makes it a more attractive technique than fresh ET.

Multiple ET increases multiple pregnancies and many related complications such as preeclampsia, prematurity, low birth weight, and perinatal mortality. A single ET can jeopardize the overall live birth rate. Balancing these risks and determining the optimum NET are particularly important.^[36]^ We observed a positive association between NET formation and the PPR. Previous studies have also shown that the Net value affects pregnancy outcomes.^[14,36]^ Pan et al demonstrated that the transfer of 2 embryos yielded a higher live birth rate than single ET.^[4]^ The results of a comprehensive meta-analysis also showed that the live birth rate after a single ET was significantly lower than that after a double ET. Moreover, single ET significantly decreases the likelihood of multiple pregnancies, whereas double ET causes an insignificant increase in multiple pregnancies.^[36]^ Conversely, Fiçicioğlu et al demonstrated that transferring 1 or 2 embryos was not related to PPR.^[37]^ Although it is known that multiple ET increase clinical pregnancy and live birth rates without significant effects on multiple pregnancies,^[36]^ it would be best to determine the optimal NET separately according to risk classifications created by considering many other factors such as age, embryo quality, and ET protocol. Therefore, comprehensive studies involving different risk groups are required.

Age is a well-known factor that affects both pregnancy and live birth rates after ET.^[4]^ Many large studies have demonstrated that oocyte quality is poor in women over 35 years of age and that the ongoing pregnancy rate and PPR are lower in this age group than in younger women. This low age-related success rate is thought to be due to an increased aneuploidy rate.^[13,35,38]^ However, the 2016 Society for ART data demonstrated no age-related reduction in implantation rates after euploid ET.^[39]^ The results of our study showed that the PPR decreased with increasing age. However, because we did not perform PGT in all the patients, the influence of chromosomal anomalies could not be conclusively identified. In a retrospective study of 22,413 IVF cycles (first autologous oocyte), there was a gradually increasing inverse correlation between pregnancy and older age (38–40, 41–42, 42+).^[13]^ In another retrospective study, increasing female age (>35 years) was shown to be one of the most important clinical factors negatively affecting pregnancy likelihood after ET.^[5]^ It seems certain that older age has a negative impact on ET success; however, it is not certain whether this effect is only due to the negative impact of older age on embryo genetics or whether other factors are at play. Therefore, we recommend that PGT be performed before ET to investigate other possible risk factors in women aged >35 years.

Improving embryo quality and identifying healthy embryos are among the most important elements of ART.^[40]^ A high incidence of aneuploidy has been associated with ET failure.^[19]^ PGT has been utilized to separate aneuploid blastocysts, thereby decreasing the risk of implantation failure and abortion, and minimizing the risk of vital chromosomal syndromes.^[41]^ In the present study, PGT levels were positively associated with PPR. In a randomized controlled trial, PGT did not result in an overall improvement in the frequency of ongoing pregnancies and live births among women aged 25 to 40 years. However, in the subgroup of women aged 35 to 40 years, there was an insignificant increase in the ongoing pregnancy rate (per ET) with the use of PGT compared with morphological embryo selection.^[19]^ As previously mentioned, this positive effect of PGT may be specific to certain age groups. Additionally, the fact that success is affected by other factors^[19]^ demonstrates the need for further research on this topic.

Our study has some limitations. As the study was retrospective, additional factors could not be studied and new data could not be added. As this was a single-center study, the generalizability of the results was limited. Differences in the number of patients between the groups (owing to the relatively high success rate) may have affected the results. An important limitation is that only early pregnancy was investigated in this study (using the β-hCG threshold). The clinical pregnancy rate, ongoing pregnancy rate live birth rate, and/or perinatal outcomes were not investigated because of the difficulty in obtaining these data. The effects of other factors that may affect pregnancy, such as infertility duration and type have not been investigated. Finally, age groups (<35 and >35 years) or responder groups were not established, limiting the stratification of our results to specific patient groups.

In conclusion, we observed that PPR decreased with age and EFD. It was also found that an increase in NET, PGT, and FET values contributed positively to the PPR. Appropriate transfer depth, ideal age, euploid embryos, FET, and higher NET can increase the PPR. However, multiple factors must be considered when deciding on the best protocol for a particular patient, including patient preference, cost, and timing. Comprehensive multicenter studies are needed to clarify the factors affecting pregnancy outcomes and the interactions between these factors.

## Author contributions

**Data curation:** Zafer Atayurt.

**Investigation:** Mustecep Kavrut, Fulya Gokdagli Sagir.

**Methodology:** Zafer Atayurt.

**Writing – original draft:** Mustecep Kavrut, Fulya Gokdagli Sagir, Zafer Atayurt.

**Writing – review & editing:** Mustecep Kavrut.
